# Climate change creates rapid species turnover in montane communities

**DOI:** 10.1002/ece3.1518

**Published:** 2015-05-25

**Authors:** Daniel K Gibson-Reinemer, Kimberly S Sheldon, Frank J Rahel

**Affiliations:** 1 Program in Ecology, University of WyomingLaramie, Wyoming; 2 Department of Zoology and Physiology, University of WyomingLaramie, Wyoming

**Keywords:** Climate change, disassembly, range shift, species’ distributions, species’ turnover

## Abstract

Recent decades have seen substantial changes in patterns of biodiversity worldwide. Simultaneously, climate change is producing a widespread pattern of species’ range shifts to higher latitudes and higher elevations, potentially creating novel assemblages as species shift at different rates. However, the direct link between species’ turnover as a result of climate-induced range shifts has not yet been empirically evaluated. We measured rates of species turnover associated with species’ range shifts in relatively undisturbed montane areas in Asia, Europe, North America, South America, and the Indo-Pacific. We show that species turnover is rapidly creating novel assemblages, and this can be explained by variable changes in species’ range limits following warming. Across all the areas we analyzed, mean species’ turnover was 12% per decade, which was nearly balanced between the loss of existing co-occurrences and the gain of novel co-occurrences. Turnover appears to be more rapid among ectothermic assemblages, and some evidence suggests tropical assemblages may be responding at more rapid rates than temperate assemblages.

## Introduction

Maintaining biodiversity and associated ecosystem processes under rapid global change presents an enormous challenge. To address this challenge, scientists and policymakers will require information on how species, assemblages, and ecosystems are being affected (Dawson et al. 2011). Shifts in species’ distributions due to climate change are well documented (Chen et al. 2011), but information regarding the impacts of these range shifts on species’ assemblages, and hence species’ interactions, has been scarce. Many ecological processes, including the regulation of population abundance, nutrient cycling, and habitat creation, are largely a result of species’ interactions within an assemblage (Chapin et al. 2000).

Previous studies of range shifts in response to climate change have focused on documenting the responses of individual species. Collectively, individual species show patterns of range shifts that are predictable: Most species shift to cooler areas. However, there is substantial variation in both the direction and the magnitude of species’ shifts. For instance, although most species shift upslope or poleward, roughly a quarter of species shift in the opposite direction and 10% show no change (Lenoir et al. 2010). Consequently, assemblages of species that previously co-occurred are not staying intact, but are rather being reshuffled as species move in different directions at different rates. This reshuffling will create new species’ interactions, as novel species’ assemblages are created.

Variation in species traits and ecosystem properties are likely to influence the impacts of climate change on range shifts and species’ assemblages. For instance, warming may have greater impacts on ectothermic animals because they have limited control of their body temperature compared to endothermic animals (Sheldon et al. 2011). Similarly, tropical areas are predicted to warm at lower rates than temperate regions, yet the response to warming may be greater in tropical species because of their narrower thermal tolerances (Dillon et al. 2010; Sheldon and Tewksbury 2014).

A major issue in climate change ecology is understanding how rising temperatures will impact the ecological processes emerging from changes in species interactions (Suttle et al. 2007; Walther 2010). A first step toward addressing this issue requires an understanding of how species assemblages are changing. A recent study showed species assemblages are changing rapidly across the world, with an estimated global mean of 10% species turnover per decade (Dornelas et al. 2014). However, it is unclear whether the observed rate of species turnover is caused by climate change or by other factors, such as invasive species or habitat loss (Dornelas et al. 2014). Our goals were to assess the rate of change in species’ assemblages caused by range shifts accompanying climate change and to identify factors that may be causing different rates of species turnover. We used published accounts of species’ range shifts in montane areas to calculate species turnover caused explicitly by range shifts. We also examined how latitude and thermal physiology affected rates of species turnover.

## Materials and Methods

### Study selection

**Figure 1:**
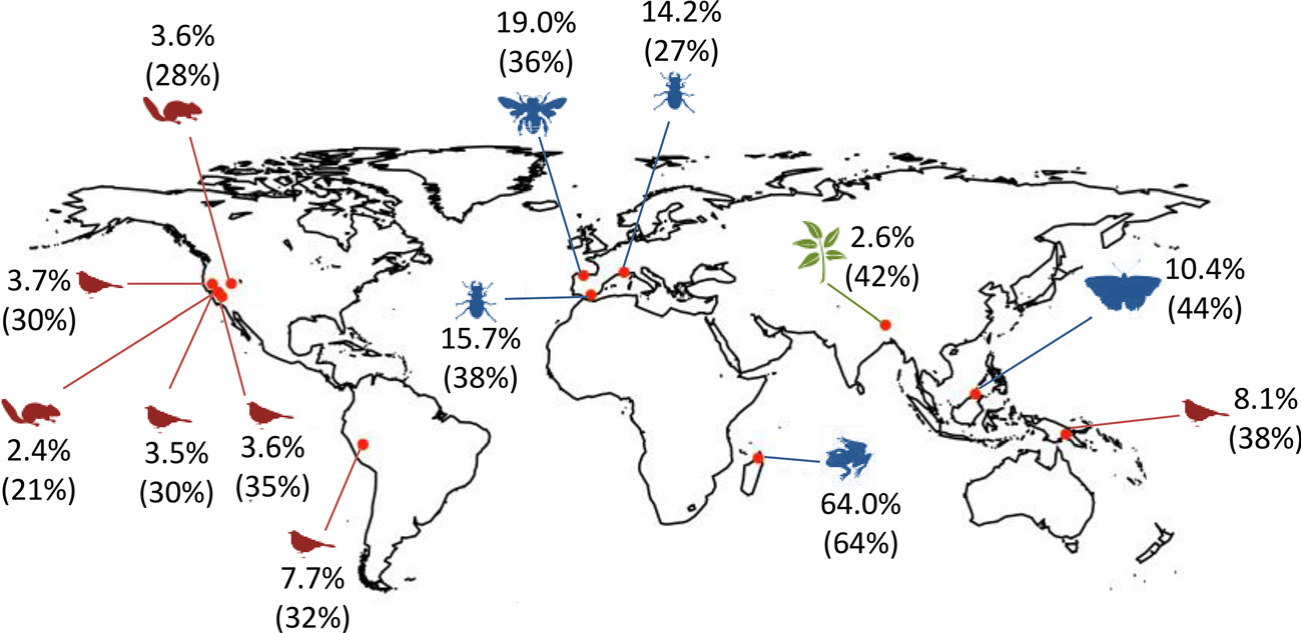
Map of study locations used in the analysis with silhouettes representing the taxonomic group measured (birds, mammals, herpetofauna, insects, and plants). Colors indicate thermal physiology guilds (red = endotherms, blue = ectotherms, green = plants). References for studies used are listed in Table1. Species turnover rates are displayed as percent turnover per decade, and absolute species turnover is listed in parentheses.

To measure the rate of species turnover along elevational gradients, we searched the literature to identify studies that had data suitable for analysis. To be included, studies had to provide information on local temperature trends and either (1) changes in both the upper and lower limits of species’ distributions in different eras, or (2) presence/absence records for fixed stations that were sampled in both historic and recent surveys. We identified published studies of range shifts associated with climate change for 716 species in 13 montane areas across four continents and three islands in the Indo-Pacific (Fig.1; Moritz et al. 2008; Raxworthy et al. 2008; Rowe et al. 2010; Chen 2011; Forero-Medina et al. 2011; Tingley 2011; Tingley et al. 2012; Ploquin et al., 2014; Telwala et al. 2013; Freeman and Freeman 2014; Menéndez et al. 2014).

Studies were generally conducted in protected areas, limiting the influence of habitat loss as a confounding factor. We relied on the assessments of the original study authors that habitat alteration was minimal between surveys. In some cases, it was possible to assess this visually, using photographs of study sites taken during historic and recent surveys (Rowe et al. 2010; Chen 2011; Moritz et al. 2008 and Tingley et al. 2012; locations available at mz.berkeley.edu/Grinnell/). Although no portion of the globe is pristine, the areas included in these studies have been among the least impacted by habitat alteration.

### Data analysis

To estimate rates of species turnover, we measured the change in species’ presence over time at fixed locations. We compared the similarity of assemblages in historic and recent surveys using presence/absence data for species. We assumed species were present within the bounds of their upper and lower limits and absent outside them. This approach is more likely to underestimate the rate of species turnover than to overestimate it and thus provides a conservative estimate of the degree of species turnover. At 100-m intervals of elevation, we calculated the temporal change in species’ assemblages using Jaccard's distance (JD), which is the complement of Jaccard's index of similarity (Dornelas et al. 2014). Jaccard's index may be vulnerable to underestimating true similarity between assemblages if there are rare, undetected species: If these species are omitted from calculations when they are actually common to both surveys, estimated turnover is inflated (Chao et al. 2005). To guard against this potential bias, we relied on species that were detected in both surveys. Less than 1% of all species in the analysis were detected in one survey but not in the other. Moreover, the data upon which these analyses are based come from studies that document a distinct pattern of species uphill range shifts, which is highly nonrandom. If false absences were driving the observed patterns of turnover, it would be highly unlikely to produce such a clear signal of uphill range shifts. Additionally, our approach is conservative with regard to present but undetected species, because we assume they are present between their upper and lower limits, which inflates estimates of similarity if a species was not detected at a point within its range.

We divided JD by study duration to standardize across studies of different durations and multiplied by 100% to produce percent species turnover per decade as follows:

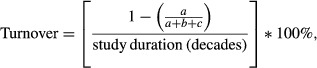
where *a* is the number of species present in both surveys; *b* is the number of species present in the first survey, but absent in the second survey; and *c* is the number of species absent in the first survey, but present in the second survey. In the same 100-m increments, we also calculated the percentage of species co-occurrences during historic surveys that were lost in recent surveys, as well as the percentage of species co-occurrences in recent surveys that were not present in historic surveys (“novel co-occurrences”). Species’ turnover values were averaged across the entire elevation gradient for each area. Using different elevation intervals (e.g., 200 m, 400 m) did not substantially influence estimates of species turnover.

A key aspect of this analysis is that range shifts from climate change can cause species turnover. Selecting studies in protected areas minimized the influence of habitat loss. We also used linear regression to test whether increased rates of range shifts led to increased rates of species turnover, using all studies that measured changes in species’ upslope range shifts. We performed linear regressions with and without a study of tropical herpetofauna that was an outlier because of the extremely high rate of species turnover (Raxworthy et al. 2008).

We compared turnover in endothermic and ectothermic assemblages after excluding one study that documented changes in plant assemblages (Telwala et al. 2013). Species turnover rates between endotherms and ectotherms were evaluated using a two-sample *t*-test and linear models. We used a two-sample *t*-test assuming equal variance with and without the outlier study of tropical herpetofauna noted above (Raxworthy et al. 2008). The interpretation of the results did not change if we excluded the outlier (*t* = −2.417, *P* = 0.036 for the test that included the outlier, and *t* = −5.868, *P* < 0.01 for the test that excluded the outlier), so we report the value of the test including the outlier. We used linear models in R (R version 3.0.2, R Development Core Team 2013) to evaluate thermal physiology (endotherm/ectotherm) as a categorical explanatory variable and absolute latitude as a continuous explanatory variable. We performed likelihood ratio tests to test the relative fit of competing models. To make sure an outlier did not drive the differences in similarity we observed between endotherms and ectotherms, we also examined linear models where the herpetofauna study was excluded. Because we found the same patterns with and without this study, we included these data in our results.

## Results

We found 719 instances in which species’ upper and lower range limits were measured in historic and recent elevational surveys, including amphibians and reptiles (*n *=* *30), birds (*n *=* *339), insects (*n *=* *177), mammals (*n *=* *49), and plants (*n *=* *124) (Table1). Across all study areas, the mean species turnover rate was 12% per decade and varied among areas (Fig.1). Considered across the duration of individual studies, the mean species turnover was 35%, and the causes of turnover were nearly balanced when averaged across studies (mean loss of co-occurrences = 36%, mean gain of novel co-occurrences = 34%, based on calculations at each elevational interval, weighted by the number of species).

**Table 1:** Studies used in the analysis of species turnover from climate change. Studies are listed in descending order by species turnover rates

Study area (Latitudinal classification)	Taxonomic group	Reference	Number of species	Species turnover per decade (%)	Absolute species turnover (%)	Co-occurrences lost/gained (%)	Study duration (years)	Warming rate (°C/decade)
Madagascar (tropical)	Reptiles and amphibians	Raxworthy et al. (2008)	30	63.9	63.9	65.7/60.5	10	0.24
Spain (temperate)	Insects	Ploquin et al. (2014)	24	19.0	36.1	31.2/47.4	19	0.45
Spain, Sierra Nevada (temperate)	Insects	Menéndez et al. (2014)	19	15.7	37.7	30.4/29.1	24	0.46
Spain, Alps (temperate)	Insects	Menéndez et al. (2014)	30	14.2	27.0	31.5/22.3	19	0.32
Borneo (tropical)	Insects	Chen (2011)	104	10.4	43.8	39.5/50.5	42	0.17
New Guinea (tropical)	Birds	Freeman and Freeman (2014)	55	8.1	38.0	30.2/41.1	47	0.08
Peru (tropical)	Birds	Forero-Medina et al. (2011)	55	7.7	31.7	31.0/37.2	41	0.19
USA, Lassen Volcanic National Park (temperate)	Birds	Tingley (2011); Tingley et al. (2012)	78	3.7	29.9	34.7/24.9	81	0.10
USA, Nevada (temperate)	Mammals	Rowe et al. (2010)	21	3.6	28.4	17.6/42.3	79	0.14
USA, Southern California (temperate)	Birds	Tingley (2011); Tingley et al. (2012)	73	3.6	35.1	47.4/26.8	98	0.08
USA, Yosemite National Park (temperate)	Birds	Tingley (2011); Tingley et al. (2012)	78	3.5	30.4	41.1/22.3	87	0.09
India (temperate)	Plants	Telwala et al. (2013)	124	2.6	42.0	42.9/35.5	159	0.14
USA, California (temperate)	Mammals	Moritz et al. (2008)	28	2.4	21.4	17.0/26.9	88	0.30

**Figure 2:**
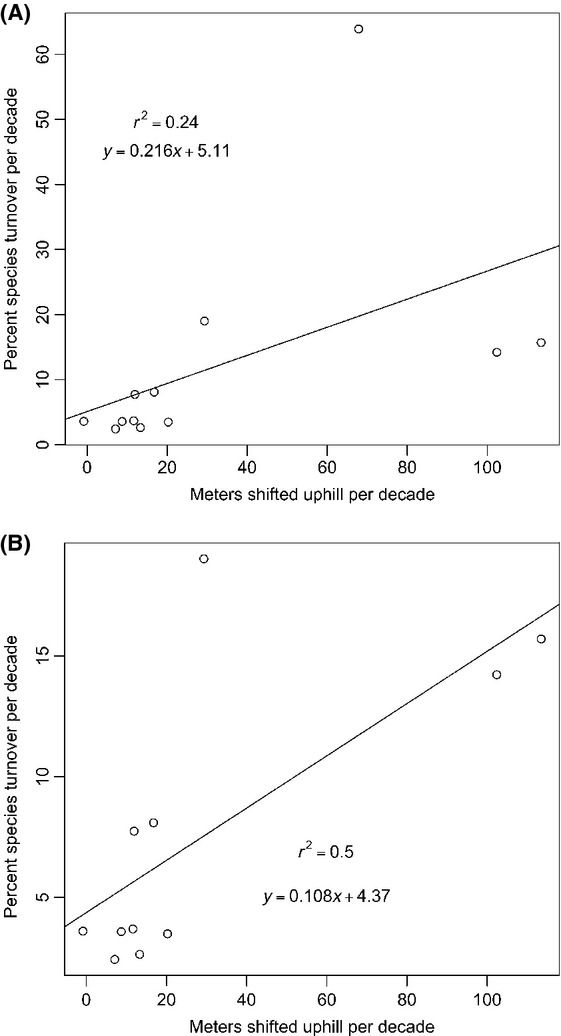
Percent species turnover per decade as a function of meters shifted upslope per decade with (A) and without (B) an outlier (Raxworthy et al. 2008).

Increased rates of species turnover appear to be caused by more rapid upslope range shifts (Fig.2). One study (Raxworthy et al. 2008) was considered an outlier because of its high rate of species turnover, but the relationship between species turnover and upslope range shifts appeared to hold with (Fig.2A) or without this study (Fig.2B).

**Figure 3:**
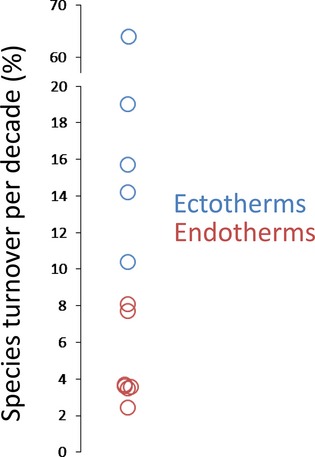
Mean rates of species turnover for endothermic and ectothermic assemblages. Turnover rates are expressed as percent per decade. Note the difference in scale on the broken *y* axis. Endotherm data points have been jittered to avoid overplotting.

The rate of species turnover varied among areas and taxonomic groups. Across all areas, the mean rate of species turnover along elevational gradients was 12% per decade; this value closely matches the recent estimate of 10% per decade for species turnover across latitudinal gradients (Dornelas et al. 2014). However, species turnover was unevenly distributed among taxa and coincided with thermal physiology. Ectothermic assemblages experienced a rate of turnover five times faster than that of endothermic assemblages (Fig.3; 25% vs. 5% turnover per decade for ectotherms and endotherms, respectively; two-sample *t*-test, *t* = −2.417, *P* = 0.04). Differences in warming rates experienced by endotherms and ectotherms cannot account for the differences in species turnover rates. The model that best predicted differences in species turnover included only thermal physiology (i.e., ectotherm vs. endotherm; linear regression, *R*^2^ = 0.37, df = 10, *P* = 0.04). The latitude term did not significantly improve model fit (*P* = 0.31).

## Discussion

In all of the assemblages we analyzed, rapid species turnover is occurring. Variable rates of climate-driven range shifts appear to be the most likely explanation for the observed species turnover. The magnitude of upslope range shifts is a good predictor of species’ turnover (Fig.2), and this makes intuitive sense: The greater the distance species move upslope, the greater the possibility that they will encounter new species and leave behind those they previously overlapped. In addition to providing a mechanistic explanation for rates of species’ turnover, climate-induced range shifts also offer more compelling explanations than other possibilities.

Alternate mechanisms that may cause species turnover in some scenarios include invasive species and random shuffling, but both are unlikely to play a substantive role in the assemblages we examined. If invasive species caused high rates of species’ turnover, they would have to constitute a large proportion of the assemblage, which is verifiably false for some assemblages (e.g., mammals in North America; Moritz et al. 2008; Rowe et al. 2010). In other cases, the invasive status of the species is less well known (e.g., moths in Taiwan; Chen 2011), but the assemblage changes that occurred could not be due invasive species because <1% of all species in the analysis were present in one survey but not the other. Thus, large range expansions due to an expanding invasion cannot account for the patterns we observed. Alternatively, species’ distributions may simply be shuffling randomly, creating species’ turnover. A previous analysis (Dornelas et al. 2014) estimated the rate of species turnover from random processes to be two orders of magnitude lower than the levels we observed, minimizing the potential for these results to be driven by random changes in species’ distributions. Additionally, if random patterns were creating the observed patterns, we would expect to see as many species shifting downslope as upslope, but each study documented a mean upslope shift. The most compelling explanation for species’ turnover in montane ecosystems is range shifts from climate change, which is sufficient to explain the observed patterns.

It is noteworthy that the loss of previous co-occurrences and the gain of novel co-occurrences are nearly balanced because this provides insight into the consequences of range shifts and species’ turnover. If species expanded their distributions upslope without losing habitat at their downslope limits, there would be many novel co-occurrences without the loss of any existing co-occurrences. Similarly, if species responded to climate change by simply retracting their distributions, there would be no novel co-occurrences. Although we measured rates of co-occurrence, not species’ interactions directly, generally species must co-occur to interact. The fact that the gains and losses of co-occurrences roughly balance is intriguing, because it suggests the number of species’ interactions may remain fairly constant even as the identity of interacting species changes.

Species turnover is not evenly distributed. In both temperate and tropical regions, ectothermic assemblages appear to be changing more quickly than endothermic assemblages (Table1 and Fig.3). It is noteworthy that species turnover rates between endotherms and ectotherms do not overlap (Fig.3). While community-level responses to climate change are an emerging field, the analyses here suggest the difference in species turnover rates between endotherms and ectotherms represents substantially different responses to climate change.

Importantly, the variation in response to climate change between ectotherms and endotherms is consistent with the knowledge of thermal physiology. The thermoneutral zone of endotherms, or the range of temperatures over which they experience low stress, may provide them with a larger cushion as temperatures increase (Khaliq et al. 2014). In contrast, the relationship between ectothermic physiology and temperature is categorically different and more nonlinear, making ectotherms more directly affected by each incremental increase in temperature (Deutsch et al. 2008). Thus, 20th-century temperature increases may have been moderate relative to the size of the thermoneutral zone for endotherms, perhaps allowing endothermic assemblages to remain more intact because ranges of endothermic species were not shifting as much as ectothermic species (Chen 2011). Additionally, endotherms may have taken greater advantage of behavioral shifts than ectotherms, such as shifting their timing of activity (Inouye et al. 2000), to remain in the same location. It is possible that the strong differences between endotherms and ectotherms we observed could have been caused by factors other than climate change, although the nonoverlapping distribution of endothermic and ectothermic assemblages suggests this possibility is unlikely.

Results of previous studies suggest that species turnover may be greater in tropical ecosystems than in temperate ecosystems (Deutsch et al. 2008; Sheldon et al. 2011). There is some support for this idea in our results. Tropical birds experienced a turnover rate more than twice that of temperate birds (a mean of 7.9% per decade for two studies involving tropical birds versus a mean of 3.6% per decade for three studies involving temperate birds, Table1). While it is not yet possible to say definitively that tropical communities are turning over faster than their temperate counterparts, the turnover rates documented here support other research highlighting the vulnerability of tropical ecosystems (Colwell et al. 2008). Although tropical regions are projected to warm less than temperate or polar regions, the impacts on tropical ectotherms will probably be more severe (Huey et al. 2009; Dillon et al. 2010). Similarly, tropical species appear to be tracking climate change more closely than temperate species (Freeman and Freeman 2014), suggesting they are more sensitive to temperature increases or that they have fewer ways to manage its impacts without moving. Several lines of evidence suggest tropical ecosystems will experience greater overall ecological impacts of climate change: Tropical ectotherms and endotherms appear to move more rapidly upslope (Freeman and Freeman 2014); tropical ectotherms experience greater metabolic stress from temperature increases (Dillon et al. 2010); and tropical assemblages appear to have higher rates of species turnover (present example).

The observed rates of species turnover far exceed rates expected from null models and involve substantial losses of species co-occurrence. Paleoecological records indicate species’ assemblages are not static, as exemplified by high turnover over several thousand years following the last glacial retreat (Williams and Jackson 2007). Yet, the levels of 20th-century community change documented here are comparable to levels of dissimilarity that have emerged over thousands of years (Williams et al. 2001). Within a century, contemporary climate change has altered species’ co-occurrences at levels more typically seen over thousands or tens of thousands of years. What happens when a large percentage of the species interactions, which generally evolve over thousands of years, are disappearing over a century? The data analyzed here are not sufficient to answer this question; however, it is likely that ecological tipping points (Scheffer et al. 2009) may be crossed when a large number of coevolved interactions break down. Similarly, recent theoretical work (Mougi and Kondoh 2012) suggests that highly diverse ecosystems are maintained primarily through the number and type of ecological interactions in the community (e.g., mutualism, competition, predator–prey, and host–parasite). The loss of co-occurring species may destabilize ecosystems where high levels of diversity are maintained through dense networks of interacting species; this may be particularly consequential for tropical assemblages, where specialization is greatest (Dyer et al. 2007).

We analyzed changes in the composition of assemblages that were mostly composed of one taxonomic group because these were the groups reported in studies. In each area, however, the entire assemblage of species consists of many interacting taxonomic groups, and many ecosystem-level processes will be influenced by species turnover across all groups. Our results show ectothermic assemblages are becoming more dissimilar than endothermic assemblages, suggesting bird and mammal composition in a given location may be fairly constant, while insect and herpetofauna composition may change rapidly. We know of only one study comparing range shifts across taxonomic groups along a single elevational transect, and this study showed significant differences in the rates of upslope shifts of plants, insects, and birds (Bässler et al. 2013). Insects were shifting faster than birds when both were measured in the same area, supporting the idea that ectothermic assemblages are more sensitive to warming than endothermic assemblages. Future studies documenting rates of species turnover across all taxonomic groups in a community are critical for understanding how the formation of novel assemblages may affect ecological processes such as plant–pollinator interactions, nutrient cycling, and disease dynamics, all of which could lead to rapid change in ecosystem services.

All studies of climate-induced range shifts face limitations stemming from the need to use the existing surveys, which necessarily restricts the geography and taxonomy of resurvey efforts. No analysis of trends across studies can overcome such limits entirely. Thus, a greater array of studies and taxonomic groups to analyze would increase the strength of inference. However, the issue of range shifts from climate change has been an urgent topic in ecology for roughly a quarter century, and an understanding of the consequences of these shifts is urgently needed for guiding conservation strategies. We analyzed data for plants, birds, mammals, beetles, bees, moths, reptiles, and amphibians from four continents and three islands. Given the inherent limitations of using existing data, at present it is simply impossible to include more species than ones in the present analysis. We hope that future studies will update this analysis as more data become available.

Although species are tracking climate change by shifting their ranges, ecological assemblages are not. Instead, assemblages are changing rapidly through a process where previously co-occurring species separate and new co-occurrences form. Novel assemblages are often predicted as a consequence of climate change, and 20th-century warming created assemblages that were substantially different over the course of decades. Importantly, this change in assemblages happened almost entirely without the extirpation of species (see Dornelas et al. 2014), demonstrating that variable shifts in elevational ranges of species are capable of producing rapid changes in assemblage composition. The consequences of such changes in assemblage composition on ecosystem function remain to be determined.

## Supporting Information

Appendix S1 Data and calculations from Chen (4).

Appendix S2 Data and calculations from Forero-Medina et al. (12).

Appendix S3 Data and calculations from Freeman and Freeman (2011).

Appendix S4 Data and calculations from Menendez et al. (18).

Appendix S5 Data and calculations from Moritz et al. (19).

Appendix S6 Data and calculations from Ploquin et al. (21).

Appendix S7 Data and calculations from Raxworthy et al. (22).

Appendix S8 Data and calculations from Rowe et al. (23).

Appendix S9 Data and calculations from Telwala et al. (2014).

Appendix S10 Data and calculations from Tingley (29) and Tingley et al. (30).
